# Sulfide (Na_2_S) and Polysulfide (Na_2_S_2_) Interacting with Doxycycline Produce/Scavenge Superoxide and Hydroxyl Radicals and Induce/Inhibit DNA Cleavage

**DOI:** 10.3390/molecules24061148

**Published:** 2019-03-22

**Authors:** Anton Misak, Lucia Kurakova, Eduard Goffa, Vlasta Brezova, Marian Grman, Elena Ondriasova, Miroslav Chovanec, Karol Ondrias

**Affiliations:** 1 Institute of Clinical and Translational Research, Biomedical Research Center, University Science Park for Biomedicine, Slovak Academy of Sciences, 845 05 Bratislava, Slovakia; anton.misak@savba.sk (A.M.); marian.grman@savba.sk (M.G.); 2 Department of Pharmacology and Toxicology, Faculty of Pharmacy, Comenius University, 832 32 Bratislava, Slovakia; lucia.k@protonmail.ch (L.K.); ondriasova@fpharm.uniba.sk (E.O.); 3 Cancer Research Institute, Biomedical Research Center, University Science Park for Biomedicine, Slovak Academy of Sciences, 845 05 Bratislava, Slovakia; eduard.goffa@savba.sk (E.G.); miroslav.chovanec@savba.sk (M.C.); 4 Faculty of Chemical and Food Technology, Slovak University of Technology in Bratislava, 812 37 Bratislava, Slovakia; vlasta.brezova@stuba.sk

**Keywords:** hydrogen sulfide, polysulfides, doxycycline, oxytetracycline, tetracycline, superoxide, hydroxyl radical, DNA cleavage, EPR spectroscopy, ^•^cPTIO radical

## Abstract

Doxycycline (DOXY) is an antibiotic routinely prescribed in human and veterinary medicine for antibacterial treatment, but it has also numerous side effects that include oxidative stress, inflammation, cancer or hypoxia-induced injury. Endogenously produced hydrogen sulfide (H_2_S) and polysulfides affect similar biological processes, in which reactive oxygen species (ROS) play a role. Herein, we have studied the interaction of DOXY with H_2_S (Na_2_S) or polysulfides (Na_2_S_2_, Na_2_S_3_ and Na_2_S_4_) to gain insights into the biological effects of intermediates/products that they generate. To achieve this, UV-VIS, EPR spectroscopy and plasmid DNA (pDNA) cleavage assay were employed. Na_2_S or Na_2_S_2_ in a mixture with DOXY, depending on ratio, concentration and time, displayed bell-shape kinetics in terms of producing/scavenging superoxide and hydroxyl radicals and decomposing hydrogen peroxide. In contrast, the effects of individual compounds (except for Na_2_S_2_) were hardly observable. In addition, DOXY, as well as oxytetracycline and tetracycline, interacting with Na_2_S or other studied polysulfides reduced the ^•^cPTIO radical. Tetracyclines induced pDNA cleavage in the presence of Na_2_S. Interestingly, they inhibited pDNA cleavage induced by other polysulfides. In conclusion, sulfide and polysulfides interacting with tetracyclines produce/scavenge free radicals, indicating a consequence for free radical biology under conditions of ROS production and tetracyclines administration.

## 1. Introduction

Endogenously produced hydrogen sulfide (H_2_S) and polysulfides (H_2_S_n_) affect many physiological and pathological processes, such as hypertension, atherosclerosis, heart failure, diabetes, inflammation, asthma, burn injuries, sepsis, angiogenesis, cancer, and neurodegenerative diseases. H_2_S and H_2_S_n_ have the beneficial effects under conditions of oxidative stress by reacting with reactive oxygen (ROS) and nitrogen (RNS) species, causing radical-induced DNA damage or possessing anti-cancer and, in some cases, pro-cancer activities [1,2,3,4,5,6,7,8,9,10,11,12,13]. H_2_S and polysulfides can interact with other cellular components, and products of these interactions have additional biological effects [3,8,14,15,16,17,18].

Since tetracyclines, in a similar manner to H_2_S/polysulfides, influence several biological processes in which free radicals or reactive species play a role, we supposed a possibility of their mutual involvement, and thus interaction, in these biological processes. Generally, tetracycline antibiotics are routinely prescribed in human and veterinary medicine to treat a wide range of infections. Doxycycline (DOXY), oxytetracycline (OXYT) and tetracycline (TETR) are used against bacterial infections through targeting bacterial ribosomes and consequently inhibiting protein synthesis [19]. DOXY is commonly used in both human and animal medicine, while OXYT and TETR are largely employed in zootechnical and veterinary practices. However, they have a variety of side effects. OXYT modulates inflammatory response [20] and TETR has an effect on growing bones and teeth [21]. DOXY induces cell death, has an anti-apoptotic function or induces apoptosis, reduces or induces ROS, triggers inflammation, reduces cardiac attack, protects cells or renal function from hypoxia-induced injury, prevents proliferation, reduces tumor growth, and suppresses a process of metastasis in human breast or prostate cancer models (for a review see [22,23,24,25,26,27]). Molecular mechanisms of these tetracyclines’ side effects are not fully understood yet and remain to be examined thoroughly.

Since ROS play an important role in many of the DOXY side effects, we have focused predominantly on H_2_S/polysulfide-DOXY interaction in connection with ROS. Since tetracyclines are widely used in clinical practice, H_2_S and polysulfides are produced endogenously and the use of H_2_S donors in medicine is being highly considered [28], we asked the following questions. Do H_2_S and/or polysulfides interact with tetracyclines (DOXY, OXYT or TETR)? Since both tetracyclines and H_2_S/polysulfides were reported to induce ROS or, in contrary, have the beneficial effects against ROS-caused damage [8,29,30,31,32,33], we wondered whether interaction of H_2_S/polysulfides with tetracyclines increase/decrease their anti-oxidant/pro-oxidant properties in terms of producing/scavenging of superoxide anion (O_2_^•−^) and hydroxyl (^•^OH) radical, as well as of reducing 2-(4-carboxyphenyl)-4,4,5,5-tetramethylimidazoline-1-oxyl-3-oxide (^•^cPTIO) radical. If H_2_S/polysulfides interact with tetracyclines, do intermediates/products of the interaction have the biological effects in vitro and *in vivo*? We found that H_2_S/polysulfides interact with tetracyclines leading to free radical producing/scavenging processes. These interactions may be the basis of the positive or undesired side effects of tetracyclines. In addition, they may be important for physiological free radical signaling, as well as for pathological conditions mediated by ROS.

## 2. Results

### 2.1. Practical Work with Polysulfides

**Figure 1:**
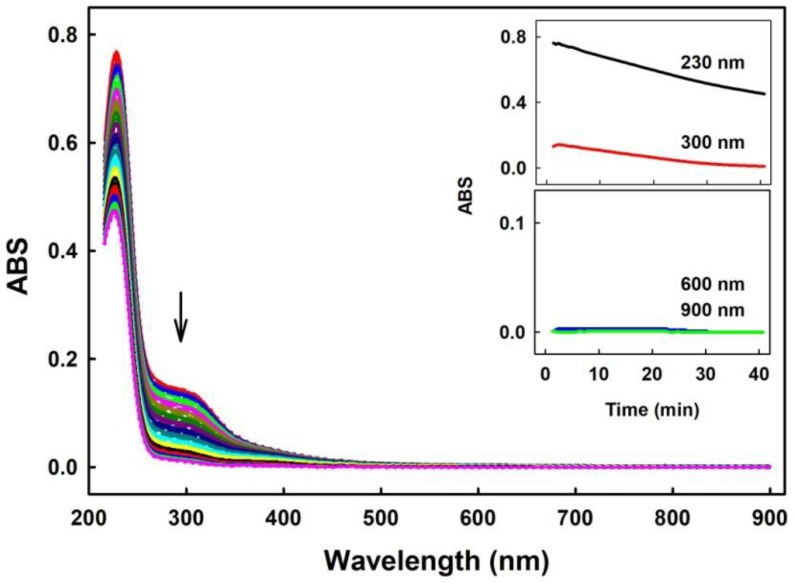
Representative time-dependent UV-VIS spectra of 100 µM Na_2_S_2_ in 100 mM sodium phosphate, 100 µM diethylenetriaminepentaacetic acid (DTPA), pH 7.4, at 37 °C. Spectra were recorded every 30 s for 40 min. The first spectrum was recorded 15 s after thawing of 10 mM Na_2_S_2_ stock. Arrow indicates decrease of ABS at 280 and 300 nm. The first spectrum is indicated by the solid red line, which is followed each 30 s by long dash red, medium dash red, short dash red, dotted red, full blue line, long dash blue, medium dash blue, etc. Insets: Kinetics of changes in ABS at 230 nm (black), 300 nm (red), 600 nm (blue) and 900 nm (green).

**Figure 2:**
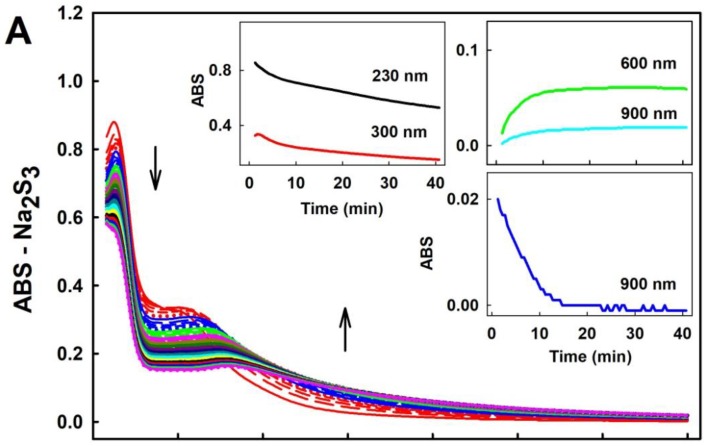

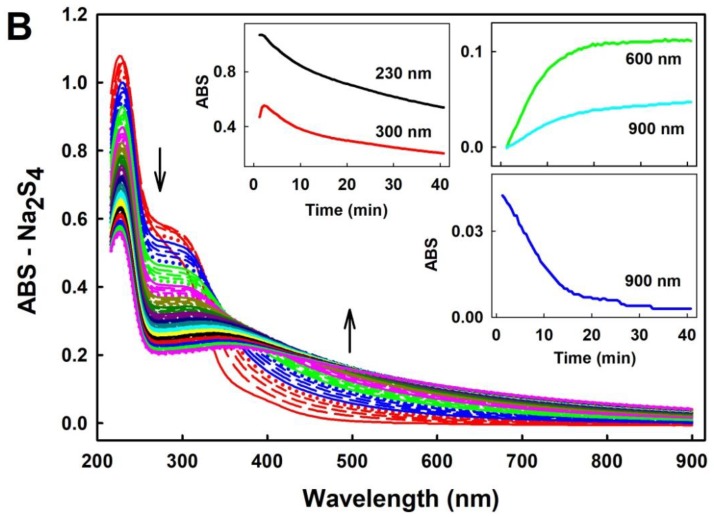
Representative time-dependent UV-VIS spectra of 100 µM Na_2_S_3_ (**A**) and 100 µM Na_2_S_4_ (**B**) in 100 mM sodium phosphate, 100 µM DTPA, pH 7.4, at 37 °C. Spectra were recorded every 30 s for 40 min. The first spectrum was recorded 15 s after thawing of 10 mM Na_2_S_3_ or 10 mM Na_2_S_4_ stocks. Arrows indicate decrease of ABS at 280 nm and increase at 500 nm. For details on colors, see Figure 1. Insets: Kinetics of changes in ABS at 230 nm (black), 300 nm (red), 600 nm (green) and 900 nm (cyan). After 40 min incubation of Na_2_S_3_ or Na_2_S_4_ samples (**A**,**B**), 100 µM ^•^cPTIO was added and the spectra were recorded for further 40 min. Kinetics of decrease of ABS at 900 nm after addition of 100 µM ^•^cPTIO (time zero) to 40 min incubated Na_2_S_3_ or Na_2_S_4_ samples are shown as Insets–blue lines.

Since polysulfides are relatively unstable in aqueous solution ([34]; for a review, see [35,36]), we have compared their stability with H_2_S under our experimental conditions. UV-VIS spectra demonstrated that the intensity of absorption peak of HS^−^ of 100–400 µM Na_2_S at 232 nm decreased by 4% within 40 min, and absorbance (ABS) at 280–300 nm (as indicator of polysulfide formation) did not change in the buffer, indicating virtually no “degradation” of H_2_S. However, UV-VIS spectra of 100 µM Na_2_S_2_, Na_2_S_3_ or Na_2_S_4_ changed over the time immediately after addition to buffer (Figure 1 and Figure 2). ABS at the region of 230 and 300 nm gradually decreased over the time for all three polysulfides, indicating degradation of the compounds. ABS at 600 and 900 nm increased for Na_2_S_3_ and Na_2_S_4_, indicating formation of undefined sulfur-containing species large enough to cause a light scattering. However, this was not the case for Na_2_S_2_, since its ABS at 600 or 900 nm was zero after 40 min (Figure 1, Inset). Notably, the increase of the ABS at 600–900 nm observed for Na_2_S_3_ and Na_2_S_4_ after 40 min incubation gradually decreased to ~0 after addition of 100 µM ^•^cPTIO to samples incubated 40 min (Figure 2, Inset-blue line). 

To demonstrate how the time-dependent degradation of polysulfides influences their biological activities, we studied their ability to reduce the ^•^cPTIO radical. The potency of Na_2_S_2_, Na_2_S_3_ and Na_2_S_4_ to reduce ^•^cPTIO was strongly time-dependent. Incubation of 100 µM Na_2_S_2_, Na_2_S_3_ or Na_2_S_4_ for 15 s followed by addition of 100 µM ^•^cPTIO caused its fast reduction in <1 min.

**Figure 3:**
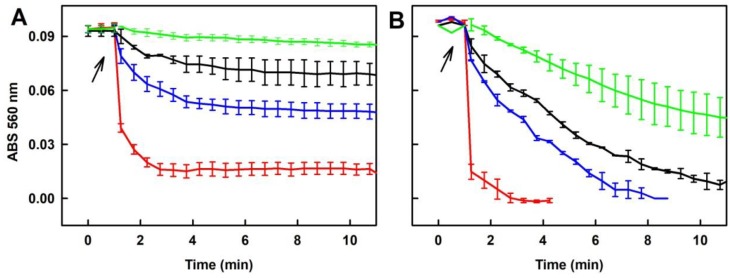
Time-dependent reduction of the ^•^cPTIO radical by Na_2_S_2_ and Na_2_S_4_. Reduction of the ^•^cPTIO radical was detected as decrease of ABS at 560 nm minus ABS at 730 nm (ABS 560 nm). Arrow indicates addition of 100 µM ^•^cPTIO to 100 µM Na_2_S_2_ (**A**) or 100 µM Na_2_S_4_ (**B**) incubated 15 s (red), 20 min (blue), 40 min (black) and 70 min (green) in the buffer consisting of 100 mM sodium phosphate and 100 µM DTPA pH 7.4, at 37 °C. Means ± SE; n = 3.

When polysulfides were incubated for 20, 40 or 70 min prior to ^•^cPTIO addition, their reducing properties markedly decreased over the time (Figure 3 and Figure S1). In case of Na_2_S_2_, they were even negligible after 70 min of incubation (Figure 3A). Extreme time-dependent instability of polysulfides in aqueous solutions should be taken into careful consideration when handling polysulfide solutions and working with exogenously added polysulfides, particularly in setting requiring a long incubation time. 

### 2.2. Formation of the O_2_^•−^ and ^•^OH Radicals by Na_2_S or Na_2_S_2_ Interacting with DOXY

**Figure 4:**
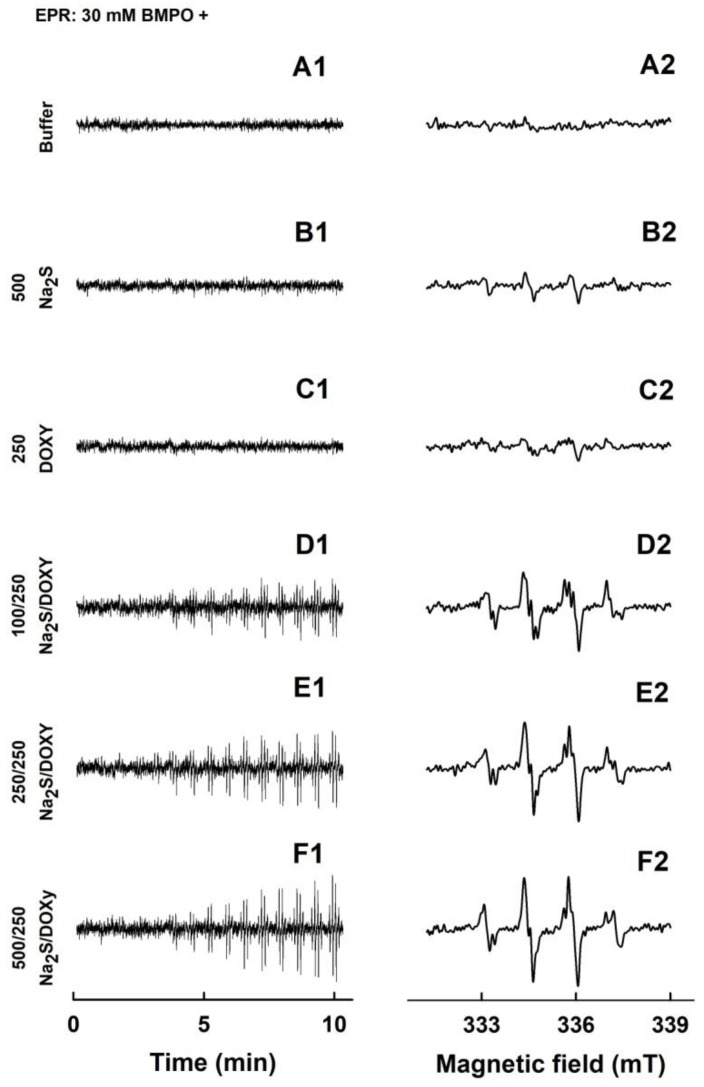
EPR spectra of the ^•^BMPO adducts for Na_2_S and DOXY and their mutual combinations. The sets of individual EPR spectra of the ^•^BMPO adducts were monitored in 15 sequential scans, each 42 s (A1–F1), starting 110 ± 15 s after sample preparation. Fifteen EPR spectra were accumulated (A2–F2). Control 30 mM ^•^BMPO (A1 and A2) and the samples containing 30 mM ^•^BMPO with 500 µM Na_2_S (B1 and B2), 250 µM DOXY (C1 and C2), the mixture of 100/250 µM/µM Na_2_S/DOXY (D1 and D2), 250/250 µM/µM Na_2_S/DOXY (E1 and E2) and 500/250 µM/µM Na_2_S/DOXY (F1 and F2). The intensities of the time-dependent EPR spectra (A1–F1) and detailed spectra (A2–F2) are comparable, as they were measured under identical EPR settings.

Since DOXY was reported to modulate in vivo biological conditions that involve ROS [24,31,32,37], we studied its interaction with radicals using EPR spectroscopy. In control spin trap experiments, 5-*tert*-butoxycarbonyl-5-methyl-1-pyrroline-*N*-oxide (BMPO) did not have EPR spectrum in the buffer solution during 11 min of observation (Figure 4A1,A2). Minor intensity of EPR spectra (mostly ^•^BMPO-OOH/OH, i.e., O_2_^•−^ and ^•^OH were trapped by BMPO) were observed when Na_2_S (500 µM) was added to BMPO (Figure 4B1,B2). This is in agreement with our previous study, in which 2 mM Na_2_S formed mostly the ^•^BMPO-OOH/OH radicals [8]. EPR spectra of the ^•^BMPO-adducts for DOXY (250 µM) were of negligible intensity only (Figure 4C1,C2), indicating that virtually no radical is produced by DOXY. However, EPR spectra could clearly be observed and their intensity increased over the time in case of the Na_2_S/DOXY mixture. At constant concentration of DOXY (250 µM), the spectral intensity of the ^•^BMPO-adducts increased over the time with the increasing concentration of Na_2_S (100–500 µM) (Figure 4D1–F2), and the ^•^BMPO-adducts spectra were stable for at least another 22 min (Figure S2A1–B2). The results revealed formation of oxygen radicals during Na_2_S/DOXY interaction.

**Figure 5:**
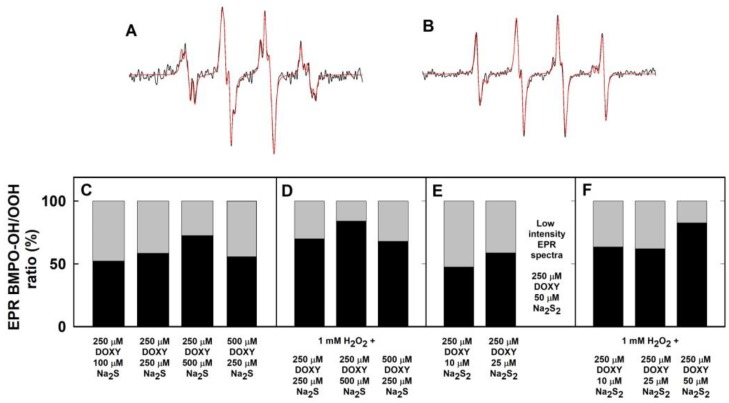
Simulation of the ^•^BMPO-adducts EPR spectra. Representative experimental (black) and simulated (red) EPR spectra of the ^•^BMPO-adducts for 250/250 µM/µM DOXY/Na_2_S (**A**) and 1 mM H_2_O_2_ with 250/500 µM/µM DOXY/Na_2_S (**B**). Magnetic field sweep 8 mT. Ratio of integral EPR spectra intensities (**C**–**F**) of simulated ^•^BMPO-OH (black) and ^•^BMPO-OOH (gray) components of the particular samples. Fifteen or thirty EPR spectra were accumulated and the hyperfine coupling constants from Table 1 were used for simulation.

To find out which radicals were trapped by BMPO, we simulated the accumulated spectra. The best fit was obtained when the hyperfine coupling constants for ^•^BMPO-OH and ^•^BMPO-OOH shown in Table 1 were used (Figure 5A,B). The constants are similar to those reported by Zhao et al. [38]. The simulation confirmed that the O_2_^•−^ and ^•^OH radicals, approximately at equimolar ratio, were formed during the Na_2_S/DOXY interaction (Figure 5C). Since the first spectrum was recorded 110 ± 15 s after sample preparation, we cannot exclude a possibility of trapping of other radicals with life-times shorter than 110 s.

**Figure 6:**
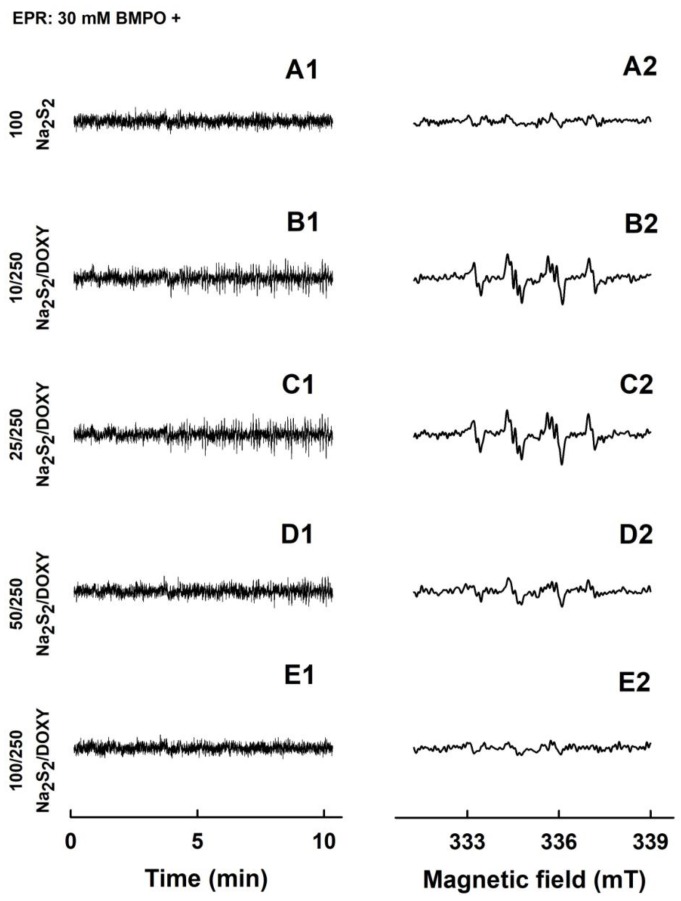
EPR spectra of the ^•^BMPO-adducts for Na_2_S_2_ and Na_2_S_2_/DOXY. Sets of individual EPR spectra of the ^•^BMPO-adducts monitored in 15 sequential scans, each 42 s (A1–E1), starting acquisition 110 ± 15 s after sample preparation. Fifteen EPR spectra were accumulated (A2–E2). BMPO (30 mM) in the presence of 100 µM Na_2_S_2_ (A1 and A2) and the mixture of 10/250 µM/µM Na_2_S_2_/DOXY (B1 and B2), 25/250 µM/µM Na_2_S_2_/DOXY (C1 and C2), 50/250 µM/µM Na_2_S_2_/DOXY (D1 and D2) and 100/250 µM/µM Na_2_S_2_/DOXY (E1 and E2). The intensities of the time-dependent EPR spectra (A1–E1) and detailed spectra (A2–E2) are comparable, as they were measured under identical EPR settings.

The EPR spectrum of the ^•^BMPO-adducts was not seen for 10, 25, 100 or 500 µM Na_2_S_2_ (Figure 6A1,A2 and Figure S2C1–E2). However, addition of DOXY to Na_2_S_2_ caused a gradual increase of the intensity of EPR spectra of the ^•^BMPO-adducts over the time (Figure 6B1–D2). Concentration-dependent EPR spectra intensities were qualitatively different for the Na_2_S_2_/DOXY and Na_2_S/DOXY mixtures. At the constant DOXY concentration (250 µM), the intensities of the ^•^BMPO-adducts were high at 10–25 µM Na_2_S_2_, decreased with increasing concentration of Na_2_S_2_, and diminished at 100 µM Na_2_S_2_ (Figure 6B1–E2). These results indicate that polysulfide Na_2_S_2_ interacting with DOXY generates oxygen radicals in a bell-shaped manner; the radicals are produced at low Na_2_S_2_ concentration (low Na_2_S_2_/DOXY molar ratio), but they are scavenged at higher Na_2_S_2_ concentrations (higher Na_2_S_2_/DOXY molar ratio). Simulated spectra revealed that O_2_^•−^ and ^•^OH were formed by the Na_2_S_2_/DOXY mixture (Figure 5E).

### 2.3. Formation of the O_2_^•−^ and ^•^OH Radicals by Na_2_S Interacting with DOXY in the Presence of Hydrogen Peroxide (H_2_O_2_)

**Figure 7:**
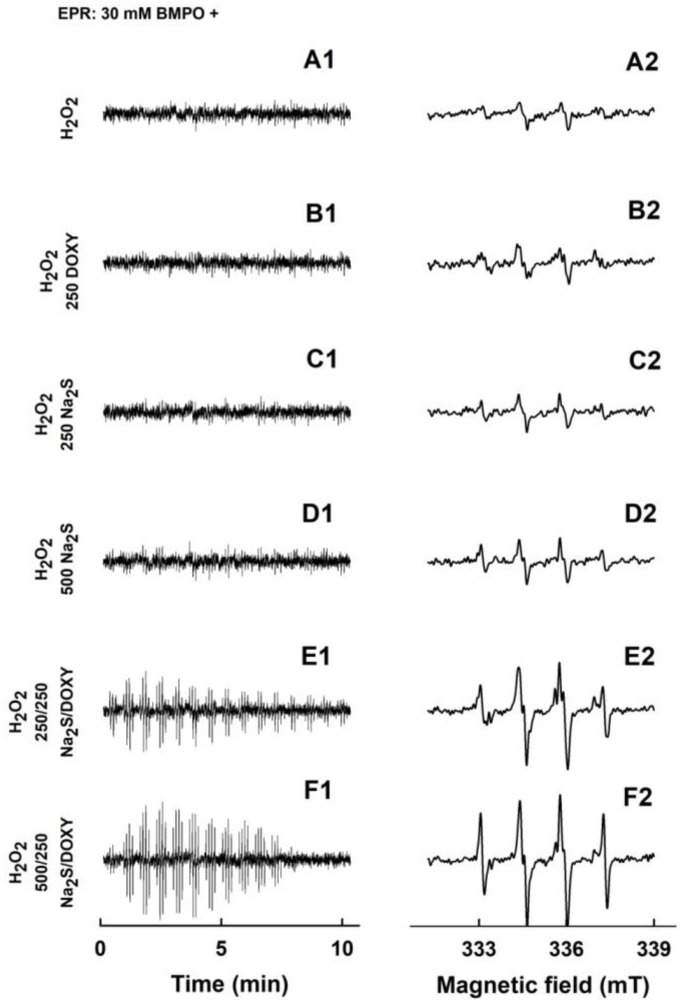
EPR spectra of the ^•^BMPO-adducts for H_2_O_2_, Na_2_S, DOXY and their mutual combinations. The sets of individual EPR spectra of the ^•^BMPO-adducts were monitored in 15 sequential scans, each 42 s (A1–F1), starting 110 ± 15 s after sample preparation. Fifteen EPR spectra were accumulated (A2–F2). Control 30 mM ^•^BMPO with 1 mM H_2_O_2_ (A1 and A2) and the samples containing 30/1 mM/mM ^•^BMPO/H_2_O_2_ with 250 µM DOXY (B1 and B2), 250 µM Na_2_S (C1 and C2), 500 µM Na_2_S (D1 and D2), the mixture of 250/250 µM/µM Na_2_S/DOXY (E1 and E2) and 500/250 µM/µM Na_2_S/DOXY (F1 and F2). The intensities of the time-dependent EPR spectra (A1–F1) and detailed spectra (A2–F2) are comparable, as they were measured under identical EPR settings.

EPR spectra of minor intensity were observed for 1 mM H_2_O_2_ (Figure 7A1,A2). Addition of 250 µM DOXY or 250–500 µM Na_2_S to 1 mM H_2_O_2_ lead to no observable change in EPR spectra (Figure 7B1–D2). However, the EPR intensity of the ^•^BMPO-adducts notably increased, when the mixture of 250/250 or 500/250 µM/µM Na_2_S/DOXY was added to 1 mM H_2_O_2_ (Figure 7E1–F2).

Bell-shaped time-dependent spectra intensities indicate an initial radical production by Na_2_S/DOXY followed by scavenging of the ^•^BMPO-adducts at the later times. Simulated spectra revealed that the O_2_^•−^ and ^•^OH radicals were formed during the Na_2_S/DOXY interaction independently of the presence of H_2_O_2_ (Figure 4, Figure 5C,D and Figure 7). Concentrations of ^•^BMPO-OH *versus* ^•^BMPO-OOH were higher in the presence of H_2_O_2_ (Figure 5C,D), however, suggesting that ^•^OH was produced by decomposition of H_2_O_2_.

### 2.4. Formation of the O_2_^•−^ and ^•^OH Radicals by Na_2_S_2_ Interacting with DOXY in the Presence of H_2_O_2_

**Figure 8:**
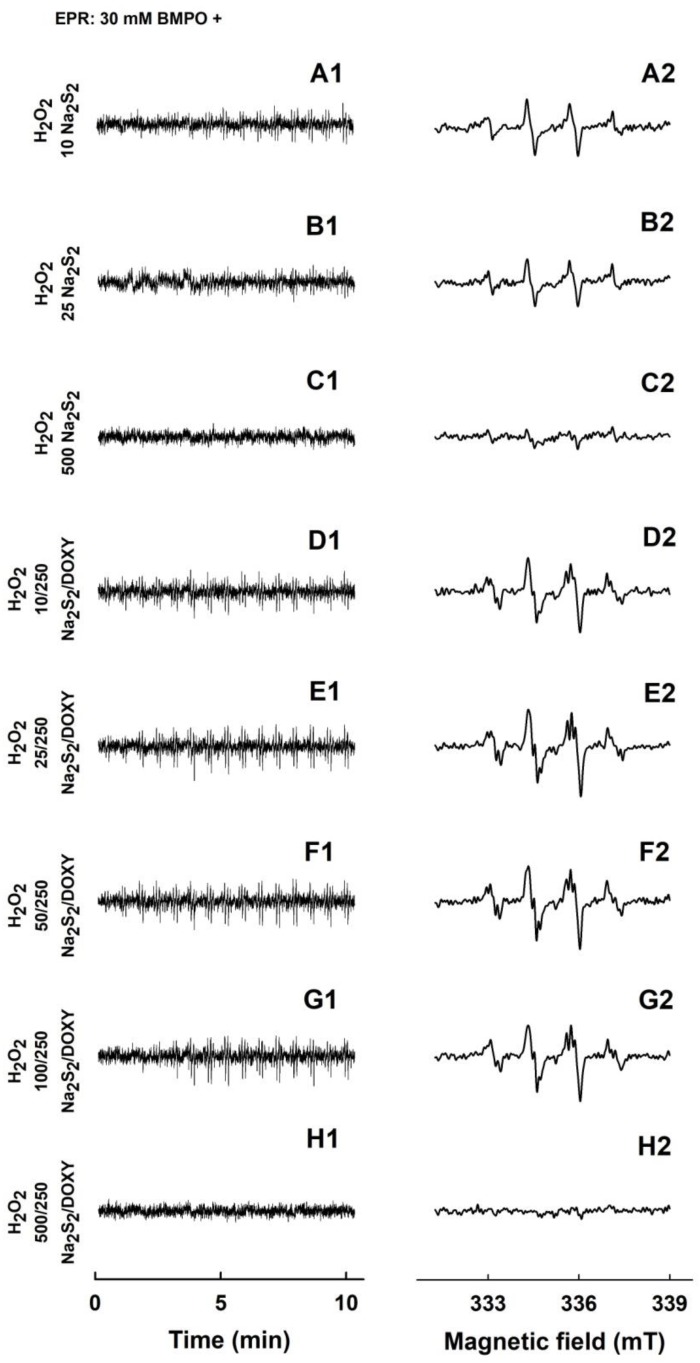
EPR spectra of the ^•^BMPO-adducts for H_2_O_2_, Na_2_S_2_, DOXY and their mutual combinations. The sets of individual EPR spectra of the ^•^BMPO-adducts were monitored in 15 sequential scans, each 42 s (A1–H1), starting acquisition 110 ± 15 s after sample preparation. Fifteen EPR spectra were accumulated (A2-H2). The samples containing 30/1 mM/mM ^•^BMPO/H_2_O_2_ with 10 µM Na_2_S_2_ (A1 and A2), 25 µM Na_2_S2 (B1 and B2), 500 µM Na_2_S_2_ (C1 and C2), the mixture of 10/250 µM/µM Na_2_S_2_/DOXY (D1 and D2), 25/250 µM/µM Na_2_S_2_/DOXY (E1 and E2), 50/250 µM/µM Na_2_S_2_/DOXY (F1 and F2), 100/250 µM/µM Na_2_S_2_/DOXY (G1 and G2) and 500/250 µM/µM Na_2_S_2_/DOXY (H1 and H2). The intensities of the time-dependent EPR spectra (A1–H1) and detailed spectra (A2–H2) are comparable, as they were measured under identical EPR settings.

Low intensity EPR spectra were observed for 1 mM H_2_O_2_ after addition of 10 or 25 µM Na_2_S_2_ (Figure 8A1–B2). This intensity even decreased upon addition of higher Na_2_S_2_ concentration (500 µM) (Figure 8C). The results indicate that Na_2_S_2_ decomposed H_2_O_2_ forming ^•^OH, which was trapped by BMPO. At high Na_2_S_2_ concentration (500 µM), it likely scavenged the formed ^•^OH and/or ^•^BMPO-OH adducts. The intensity of the ^•^BMPO-adducts noticeably increased, when the mixture of Na_2_S_2_/DOXY 10/250, 25/250, 50/250 and 100/250, but not 500/250 µM/µM, was added to 1 mM H_2_O_2_ (Figure 8D1–H2). This production of the radicals at low Na_2_S_2_/DOXY ratio and scavenging them at high(er) ratio indicates concentration-dependent bell-shaped production/inhibition of the radicals. Simulated spectra revealed that the O_2_^•−^ and ^•^OH radicals were produced by Na_2_S_2_/DOXY in the presence of H_2_O_2_ (Figure 5F).

### 2.5. Potency of Compounds to Reduce the ^•^cPTIO Radical

In the previous section, we showed that Na_2_S and Na_2_S_2_ interacting with DOXY possess different time- and concentration-dependent potency to produce and scavenge radicals. Since the Na_2_S/DOXY/H_2_O_2_ and Na_2_S_2_/DOXY mixtures behave in the bell-shaped manner in these reactions, initially producing radicals followed by scavenging them, we next studied the potency of Na_2_S/DOXY and Na_2_S_2_/DOXY to reduce the ^•^cPTIO radical as a model radical system. Decrease of ^•^cPTIO ABS at 560 nm was used to measure reduction of the ^•^cPTIO radical. We compared the effect of DOXY with other tetracycline derivatives, OXYT and TETR, and antibiotics, fusaric acid (FUSA) and norfloxacin (NORF), to reduce the ^•^cPTIO radical alone and in combination with Na_2_S and polysulfides. For this purpose, we extended the set of studied polysulfides with Na_2_S_3_ and Na_2_S_4_.

#### 2.5.1. Tetracyclines, but neither FUSA nor NORF, Reduce the ^•^cPTIO Radical in the Presence of Na_2_S

**Figure 9:**
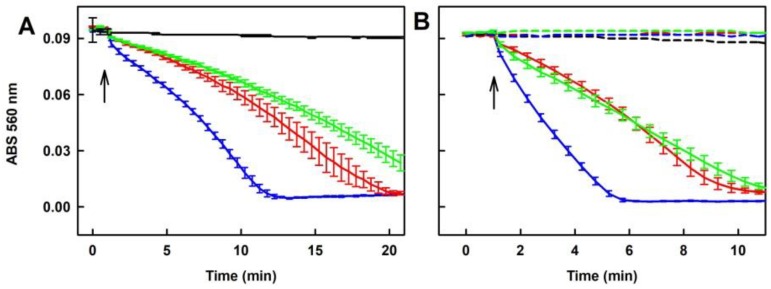
Time-dependent reduction of the ^•^cPTIO radical by the studied compounds. Reduction of the ^•^cPTIO radical was detected as decrease of ABS at 560 nm minus ABS at 730 nm (ABS 560 nm). Buffer: 100 mM sodium phosphate, 100 µM DTPA, pH 7.4, at 37 °C. Arrow indicates addition of Na_2_S or/and tetracyclines to 100 µM ^•^cPTIO. (**A**) Na_2_S (400 µM) added to ^•^cPTIO (black); Na_2_S (400 µM) added to ^•^cPTIO containing 50 µM (green), 100 (red) and 200 µM (blue) DOXY. (**B**) Comparison of time-dependent reduction of ^•^cPTIO (100 µM) by 200 µM Na_2_S (dash black), 400 µM DOXY (dash red), 400 µM OXYT (dash green), 400 µM TETR (dash blue) alone and after addition of the Na_2_S/DOXY (red), Na_2_S/OXYT (green) or Na_2_S/TETR (blue) mixtures (200/400 μM/μM). Means ± SE, n = 2–5.

Na_2_S (400 µM) reduced ^•^cPTIO (100 µM) by <1% after 20 min (Figure 9A). DOXY, OXYT and TETR, on their own had only minor effects in this context (Figure 9B). However, in the presence of Na_2_S they reduced ^•^cPTIO. The reducing potency of the mixture increased with increasing concentration of DOXY (50–200 µM; Figure 9A). When the potency of the mixtures of DOXY, OXYT or TETR (400 µM) with Na_2_S (200 µM) was compared, the following order was obtained: Na_2_S/TETR>Na_2_S/DOXY~Na_2_S/OXYT>>Na_2_S~0 (Figure 9B). It was of interest to know if fluoroquinolone antibiotic NORF (400 µM) or fungal toxin FUSA (400 µM) can reduce ^•^cPTIO. These compounds *per se* or in the presence of Na_2_S (400 µM) had minor effects, as they reduced ^•^cPTIO <4% after 20 min (Figure S3).

#### 2.5.2. Ability of the Polysulfide/Tetracyclines Mixture to Reduce the ^•^cPTIO Radical

**Figure 10:**
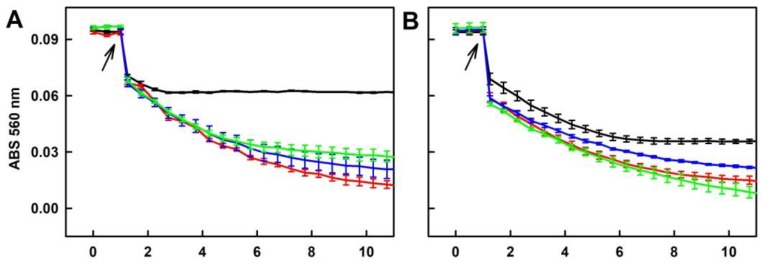

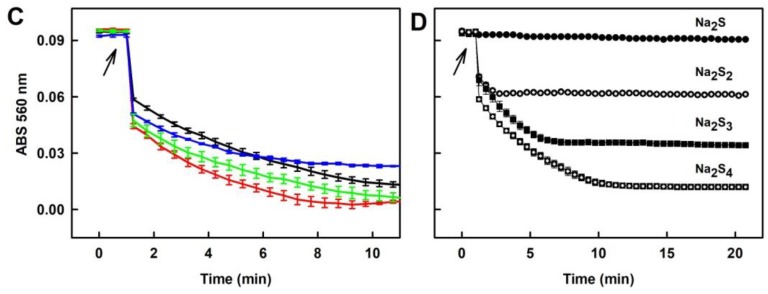
Time-dependent reduction of ^•^cPTIO by the polysulfide/tetracyclines interaction. Kinetics of changes in ABS at 560 nm minus 730 nm (ABS 560 nm) of 100 µM ^•^cPTIO after addition (indicated by arrow) of 40 µM Na_2_S_2_ (**A**), Na_2_S_3_ (**B**) and Na_2_S_4_ (**C**) (black lines) and their mixtures with 400 µM DOXY (red line), OXYT (green line) and TETR (blue line). The comparison of time-dependent potency of 40 µM Na_2_S (full circles), Na_2_S_2_ (open circles), Na_2_S_3_ (full squares) and Na_2_S_4_ (open squares) to reduce 100 µM ^•^cPTIO (**D**).

Since polysulfides Na_2_S_2_, Na_2_S_3_ and Na_2_S_4_ at 100 µM concentration reduced ^•^cPTIO in <1 min (Figure 3), we used lower 40 µM concentrations to study their effects in a mixture with tetracyclines. All tetracyclines potentiated ability of Na_2_S_2_ and Na_2_S_3_ to reduce ^•^cPTIO (Figure 10A,B). In case of Na_2_S_4_, the effects were less pronounced (Figure 10C). It is noteworthy that the extent and rate of the polysulfides’ ability to reduce ^•^cPTIO depends on an amount of sulfurs atoms. Efficiency of Na_2_S, Na_2_S_2_, Na_2_S_3_ and Na_2_S_4_ (40 µM) to reduce ^•^cPTIO (100 µM) was 0%, 35%, 63% and 87% respectively, and the rate of reduction might be different depending on sulfur atoms (Figure 10D).

### 2.6. Tetracyclines Cleave pDNA in the Presence of Na_2_S, but Inhibit pDNA Cleavage Induced by Polysulfides

**Figure 11:**
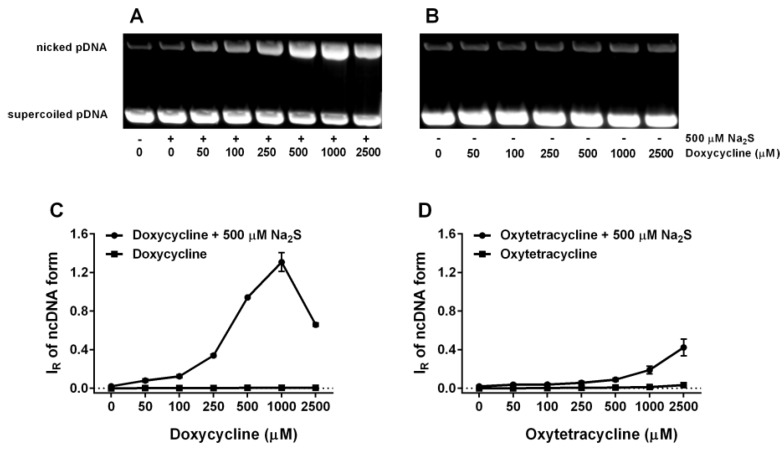

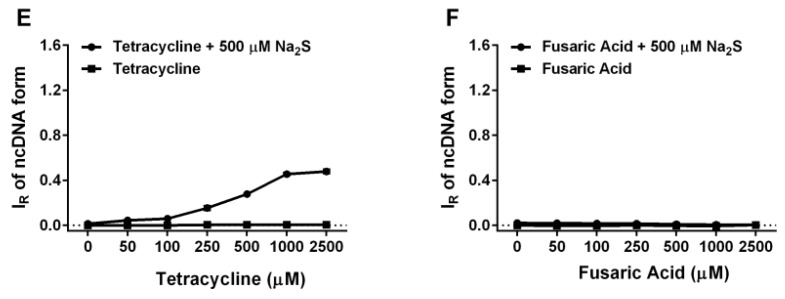
pDNA cleavage potency of tetracyclines in the presence of Na_2_S. Representative gels demonstrating the effect of DOXY on the pDNA integrity in the presence (**A**) and absence of 0.5 mM Na_2_S (**B**) are shown. The bands at the bottom correspond to the circular supercoiled form of pDNA and the more or less intense bands appearing above it represent nicked circular pDNA. Quantitative representation of the concentration-dependent effect of DOXY (**C**), OXYT (**D**), TETR (**E**) and FUSA (**F**) on pDNA integrity in the presence (circle) and absence (square) of 0.5 mM Na_2_S. Means ± SE, n = 3.

**Figure 12:**
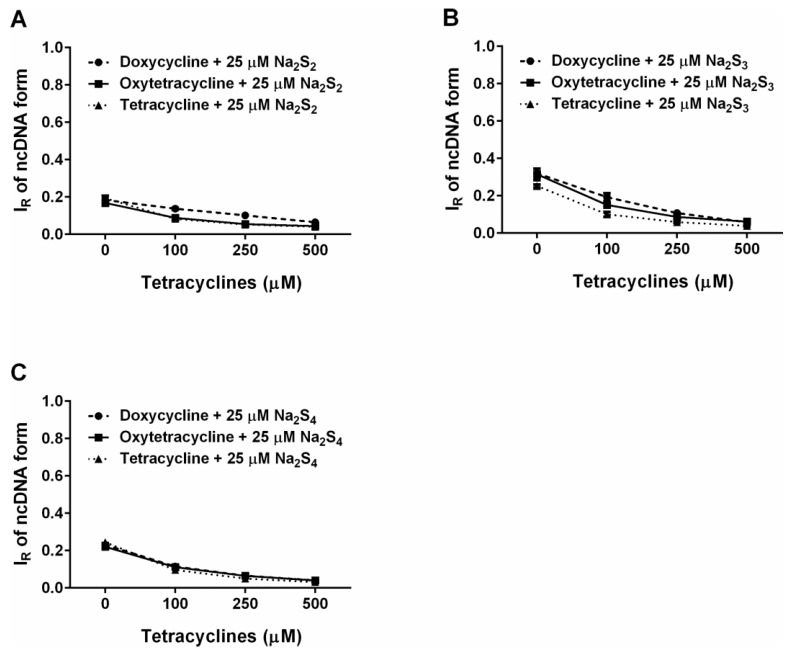
Inhibiting effects of the tetracyclines/polysulfides interaction on polysulfide-induced pDNA cleavage. Concentration-dependent inhibiting effect of DOXY (circle), OXYT (square) and TETR (triangel) in the presence of 25 µM Na_2_S_2_ (**A**), Na_2_S_3_ (**B**) and Na_2_S_4_ (**C**) on pDNA cleavage. Means ± SE, n = 3. For the tetracyclines’ effects on their own, see Figure 11.

To put into the biological frame our findings on free radical producing/scavenging interaction between tetracyclines and reactive sulfur species, which seem to be time- and concentration-depended, we used well-characterized radical-induced pDNA cleavage assay. Tetracyclines (0.05–2.5 mM) alone have virtually no pDNA damaging effects. However, in the presence of Na_2_S (0.5 mM) the cleavage potencies robustly increased in the following order: DOXY > TETR ≥ OXYT >> FUSA ~ 0 (Figure 11). Interestingly, the Na_2_S/DOXY mixture exhibited the pDNA damaging effects with the bell-shaped characteristics. NORF was not used in this study due to low solubility in the reaction buffer at the listed concentrations. All studied polysulfides slightly cleaved pDNA similarly to our previous findings on Na_2_S_4_ [8]. However, tetracyclines (DOXY, OXYT and TETR) in the presence of the polysulfides inhibited their pDNA cleavage potency in the concentration-dependent manner (Figure 12).

### 2.7. Na_2_S Did Not Modify Inhibitory Effect of DOXY on Growth of Escherichia Coli Cells

**Figure 13:**
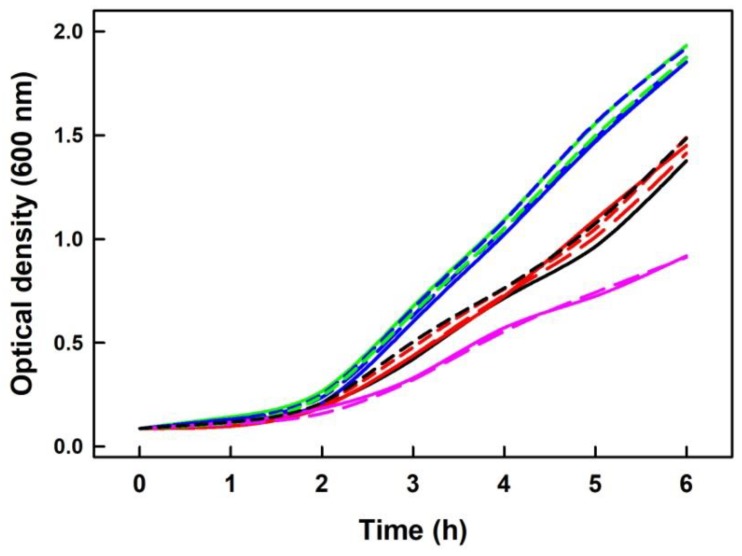
The effects of Na_2_S on bacterial growth in the presence of DOXY. Representative growth curves of parallel samples derived from three independent experiments show no significant effects of Na_2_S on *E. coli* cells undergoing DOXY treatment. Control (n = 2; dash green, green), 50 nM DOXY (n = 2; dash black and black), 10 µM Na_2_S (short dash blue), 25 µM Na_2_S (long dash blue), 50 µM Na_2_S (blue), 50/10 nM/µM DOXY/Na_2_S (short dash red), 50/25 nM/µM DOXY/Na_2_S (long dash red), 50/50 nM/µM DOXY/Na_2_S (red), 100/50 nM/µM DOXY/Na_2_S (dash pink) and 100 nM DOXY (pink). Growth of bacterial culture was measured as change in optical density at 600 nm (OD**_600_**) within six hours.

To examine whether exogenously added Na_2_S has an observable effect on living cells undergoing DOXY treatment, we measured the growth of *E. coli* cells in the presence of DOXY and several concentrations of Na_2_S. Growth of bacterial culture was measured as change in the optical density (OD) at 600 nm within six hours. As shown (Figure 13), the presence of exogenous source of Na_2_S had no significant effect on bacterial cells treated with 50 nM or 100 nM DOXY.

## 3. Discussion

### 3.1. Practical Use of Polysulfides versus Na_2_S

Our study confirmed and underlined that polysulfides (or their anion form) relative to Na_2_S are unstable in buffer solutions at 37°C and their effects strongly depend on time of their storage or incubation (Figure 1, Figure 2 and Figure 3). This should be taken into careful consideration particularly in a long(er) time experimental settings. The exact species formed by polysulfides in the aqueous solutions are still unclear [35]. Based on the differences in the time-dependent UV-VIS spectra (ABS at 600 and 900 nm; Figure 1 and Figure 2), we suggest that different species are formed from Na_2_S_2_ and Na_2_S_3_ or Na_2_S_4_. ABS of HS^–^ has a peak at 232 nm. Since ABS at 232 nm decreased for all polysulfides over the time, we suggest that during the polysulfides incubation no, or negligible, amount of HS^–^ molecules are formed (Figure 1 and Figure 2, Insets). The decrease of ABS at 600–900 nm by ^•^cPTIO added to 40 min of incubated Na_2_S_3_ and Na_2_S_4_ solutions indicates disturbing of the light scattering sulfur-containing species by ^•^cPTIO. Since decrease of ABS at 900 nm (Figure 2, Inset-blue line) correlated with the decrease of the ^•^cPTIO radical concentration (Figure 3B, black line), we may assume that electron transfer from the light scattering sulfur-containing species to ^•^cPTIO occurs.

### 3.2. Interaction of Na_2_S and Polysulfides with Tetracyclines

Here, we provide the first evidence that Na_2_S and polysulfides interact with DOXY and that this interaction produces/scavenges the O_2_^•−^ and ^•^OH radicals. Mechanistic details of these interactions remain unknown yet, but we propose that the Na_2_S/DOXY and Na_2_S_2_/DOXY product(s) may transfer electron to oxygen forming O_2_^•−^, which can be trapped by BMPO and detected as ^•^BMPO-OOH (Figure 4 and Figure 6). The EPR spectra detecting ^•^BMPO-OH confirm that ^•^OH can also be trapped by BMPO. It cannot be excluded that a certain part of ^•^BMPO-OH was caused by decomposition of ^•^BMPO-OOH to ^•^BMPO-OH by reduction properties of Na_2_S/DOXY and Na_2_S_2_/DOXY. ^•^BMPO-OH was observed after Na_2_S/DOXY or Na_2_S_2_/DOXY interaction in the presence of H_2_O_2_. We propose that ^•^OH trapped by BMPO was produced by decomposition of H_2_O_2_ caused by the Na_2_S/DOXY and Na_2_S_2_/DOXY mixtures (Figure 7 and Figure 8). In our previous study, Na_2_S_4_ was more potent scavenger of radicals and producer of ^•^OH by decomposition of H_2_O_2_ compared to Na_2_S [8]. Our present results indicate that Na_2_S_2_ and Na_2_S_4_ possess similar properties in this manner.

The bell-shaped production of O_2_^•−^ and ^•^OH and scavenging of the ^•^BMPO-OOH/OH radicals by increasing ratio of Na_2_S_2_/DOXY (Figure 6) can be explained by reduction potency of high concentration of Na_2_S_2_, as documented by reduction of the ^•^cPTIO radical, which is potentiated by DOXY (Figure 10A). At high Na_2_S_2_/DOXY ratio (100/250 µM/µM), ^•^BMPO-OOH/OH spectra were not detected due to reduction of radicals during Na_2_S_2_/DOXY interaction and/or reduction of ^•^BMPO-OOH/OH. In our previous study, we reported that Na_2_S_4_ possesses higher potency to reduce ^•^cPTIO than Na_2_S [8]. In the present study, we found high ^•^cPTIO reducing properties, being comparable to that of Na_2_S_4_, for other two polysulfides, Na_2_S_2_ and Na_2_S_3_ (Figure 3 and Figure 10). Based on comparison of the time-dependent ability of Na_2_S and polysulfides to reduce ^•^cPTIO (Figure 10D), we may assume that the effectiveness in reducing of the ^•^cPTIO radical depends on sulfur(s) chemical configuration/arrangement in H_2_S_n_ (n ≥ 1) species.

Several studies showed that tetracyclines produce and inhibit radicals in different, mostly non-physiological conditions. Tetracyclines during photochemical oxidation, autoxidation or oxidation with a Fenton reagent in the aqueous solution produced O_2_^•−^, ^•^OH, H_2_O_2_ or singlet oxygen [39,40,41,42]. In addition, DOXY can induce ROS production [43]. Kladna et al. found that in dimethyl sulfoxide DOXY and OXYT generated O_2_^•−^ at low concentrations, but scavenged it at high concentrations [42]. However, under our experimental conditions and by using EPR spectra of spin trap BMPO, we could detect formation of the O_2_^•−^ and ^•^OH radicals only in the mixture of DOXY/Na_2_S or DOXY/Na_2_S_2_. Based on our data, we speculate that sulfide and polysulfide interacting with tetracyclines may modulate O_2_^•−^ redox chemistry.

Here, we provide evidence that H_2_S and polysulfides interacting with tetracyclines reduce ^•^cPTIO and modulate cleavage of pDNA. Such properties are not generally adopted by all antibiotics, since FUSA and NORF do not notably interact with Na_2_S, based on their ^•^cPTIO reduction inefficiency. In addition, FUSA do not damage pDNA alone or in the presence of Na_2_S. Hence, it can be proposed that radicals produced during Na_2_S/polysulfides-tetracyclines interaction (Figure 4, Figure 5, Figure 6, Figure 7 and Figure 8) are involved in pDNA cleavage (Figure 11) and that high reduction properties of the polysulfides/tetracyclines mixtures in comparison to Na_2_S/tetracyclines mixtures are responsible for the inhibitory effects of tetracyclines in the presence of polysulfides (Figure 12). The polysulfides/tetracyclines mixtures can efficiently scavenge radicals before they reach and damage pDNA. Recently, Gallo et al. reported that OXYT induces DNA damage, the fact representing a possible risk for human and animal health [44]. In contrast, no pDNA damage caused by OXYT on its own was observed under our experimental conditions. Only in the mixture with Na_2_S, OXYT was able to induce DNA injury (Figure 11D).

It was proposed that bactericidal activities of tetracyclines may results from their capability of producing ROS, and involvement of ROS in bactericidal activities has become the subject of extensive debate [33]. Therefore, it was of high priority to know if radicals produced during Na_2_S/DOXY interaction influence the antibacterial effects of DOXY. Since addition of Na_2_S has no significant effect on bacterial cells growing in the presence of DOXY (Figure 13), it could be supposed that radicals produced during Na_2_S/DOXY interaction do not have any important influence in this type of in vivo experiment at the given Na_2_S/DOXY concentrations. However, a complexity of in vivo system may rather mask existence, extend and contribution of certain reactions, particularly if they are backed up, to the resulting phenotype and additional more sophisticated experimental setup is required to adequately address this issue. One of the options would be to use strains that can produce H_2_S endogenously so that its steady-state levels are ensured throughout whole experiment.

To our best knowledge, there is no kind of information on interaction of DOXY with H_2_S or polysulfides. Numerous qualitatively and quantitatively different time- and concentration-dependent data, which we provide, indicate that the H_2_S/polysulfides-tetracyclines interactions are highly complex, and specific additional “chemical” approach is therefore needed to delineate them.

### 3.3. Possible Biological Consequences of the Na_2_S and Polysulfides Interacting with Tetracyclines

Maximum human plasma concentrations of DOXY are usually ranging from 1.5 to 7.0 µg/mL or from 3 to 14.6 µM [45,46]. Concentrations of endogenously produced polysulfides in HeLa cells are ~0–120 nM [47] and H_2_S concentration can be higher than 1 µM [48]. However, in the very local environment presence of 1 molecule in 1000 nm^3^ gives raise of 1.65 mM H_2_S or polysulfide concentration. We assume that H_2_S and polysulfide concentrations might be time-dependently higher *in situ*, where they are enzymatically produced and/or released from intracellular H_2_S stores [1]. Another source of H_2_S could be H_2_S donors, which are extensively studied as H_2_S releasing drugs [3,28]. The mutual administration of H_2_S donors with tetracyclines is challenge for the future studies and can contribute to understanding of a biological relevancy of the H_2_S/polysulfide-tetracycline interactions.

Tetracyclines have several positive and negative biological effects in which free radicals might play a role. For example, DOXY protects human intestinal cells or renal function from hypoxia/reoxygenation injury, improves cardioprotection [24,32,37], protects against ROS-induced mitochondrial fragmentation and isoproterenol-induced heart failure [31], inhibits mitochondrial biogenesis and alters energy metabolism [49,50]. DOXY induces cell death and prevents the proliferation of several types of cell by inducing ROS production [43]. OXYT induces oxidative damage in liver and kidney [51,52], oxidative stress and immunosuppression in rainbow trout [53], and modulates inflammation, apoptosis and cancer [54,55,56,57]. Whether and how H_2_S/polysulfides-tetracycline interaction plays a role in these biological effects is a challenge for the future research.

## 4. Materials and Methods

### 4.1. Chemicals

10 mM stock solutions of the studied compounds, doxycycline hydrochloride (DOXY; D3447 Merck, Bratislava, Slovakia), oxytetracycline hydrochloride (OXYT; O5875 Merck, Bratislava, Slovakia), tetracycline hydrochloride (TETR; T7660, Merck, Bratislava, Slovakia) and fusaric acid (FUSA; F6513 Merck, Bratislava, Slovakia), were prepared in deionized H_2_O and used within ≤6 h. 10 mM stock solution of norfloxacin (NORF; N9890 Merck, Bratislava, Slovakia) was dissolved in DMSO by 1 min bath sonication and used within ≤6 h. Na_2_S as a source of H_2_S (100 mM; SB01, DoJindo, Munich, Germany) and polysulfides, sodium disulfide (Na_2_S_2_, 10 mM), sodium trisulfide (Na_2_S_3_, 10 mM) and sodium tetrasulfide (Na_2_S_4_, 10 mM) (SB02, SB03 and SB04, SulfoBiotics, DoJindo, Munich, Germany), were prepared in argon-bubbled deionized H_2_O, aliquoted, stored at −80 °C and thawed just before the use. Na_2_S dissociates in solution and reacts with H^+^ to yield H_2_S, HS^−^ and a trace of S^2−^. We use the term Na_2_S to encompass the total mixture of H_2_S, HS^−^ and S^2−^. Similarly, Na_2_S_2_, Na_2_S_3_ and Na_2_S_4_, dissociate in solution yielding S_n_^2−^, HS_n_^−^ and traces of H_2_S_n_ (n = 2–4). For simplicity, we again use terms Na_2_S_2_, Na_2_S_3_ and Na_2_S_4_. The radical ^•^cPTIO (10 mM, 81540 Cayman, Neratovice, Czech Republic or C221, Merck, Bratislava, Slovakia) prepared in deionized H_2_O was stored at −20 °C for several weeks. 100 mM sodium phosphate buffer supplemented with 100 µM DTPA, pH 7.4, 37 °C, was employed for UV-VIS experiments.

### 4.2. EPR of the ^•^BMPO-adducts

To study an involvement of radicals in Na_2_S/polysulfides/DOXY interaction, EPR study of spin trap BMPO was used and conducted in accordance with previously reported protocols [8]. To the solution (final concentrations) of BMPO (30 mM), DTPA (100 µM) in sodium phosphate buffer (50 mM, pH 7.4, 37 °C), aliquots of the compounds were added. The sample was mixed for 5 s and transferred to a standard cavity aqueous EPR flat cell. The first EPR spectrum was recorded 110 ± 15 s after mixing the sample. The sets of individual EPR spectra of the ^•^BMPO spin-adducts were recorded as 15 sequential scans, each 42 s, within a total time of 11 min. EPR spectra of the ^•^BMPO spin-adducts were measured on an EMX spectrometer (Bruker, Rheinstetten, Germany) X-band ~9.4 GHz, 335.15 mT central field, 8 mT scan range, 20 mW microwave power, 0.1 mT modulation amplitude, 42 s sweep time, 20.48 ms time constant, and 20.48 ms conversion time at 37 °C.

### 4.3. UV-VIS of ^•^cPTIO

To 900–990 µL solution of 100 mM sodium phosphate, 100 µM DTPA buffer (pH 7.4, 37 °C), the final concentrations of the studied compounds were added (final volume 1 mL). UV-VIS spectra (900–190 nm) were recorded 40 (80) × 30 s using a Shimadzu 1800 spectrometer (Kyoto, Japan) at 37 °C (blank was H_2_O). For our study, the ^•^cPTIO extinction coefficient at 560 nm of 930 M^−1^ cm^–1^ was used. Scavenging of the ^•^cPTIO radical by the studied compounds was determined as a decrease of ABS at 560 nm (absorption maximum of ^•^cPTIO) minus ABS at 730 nm after subtraction of baseline absorbance [8].

### 4.4. pDNA Cleavage Assay

The pBR322 plasmid (N3033L, New England BioLabs Inc., Frankfurt, Germany) was used in pDNA cleavage assay that was performed according to our previous report [8]. In this assay, all samples contained 0.2 µg pDNA in a sodium phosphate buffer (25 mM sodium phosphate, 50 µM DTPA, pH 7.4, 37 °C). After addition of compounds, the resulting mixtures were incubated for 30 min at 37 °C. Afterwards, the reaction mixtures were subjected to 0.6% agarose gel electrophoresis. The samples were electrophoresed in TBE buffer (89 mM Tris, 89 mM boric acid, 2 mM EDTA, pH 8.0) at 5.5 V/cm for 2 h. Gels were stained with Gel Red™Nucleic Acid Gel Stain and photographed using the Odyssey Fc Imaging System (LI-COR Biotechnology, Bad Homburg, Germany). The integrated densities of two identified pBR322 forms (supercoiled and nicked circular form) in each lane were quantified using Image Studio analysis software (LI-COR Biotechnology, Bad Homburg, Germany) to estimate pDNA cleavage efficiency.

### 4.5. Bacterial Growth Measurement

LB medium (1% bacto tryptone, 1% NaCl, 0.5% yeast extract, pH 7.0) was inoculated by a single colony of E. coli strain RRI (F^−^ *mcr*B *mrr hsd*S20(r_B_^−^, m_B_^−^) *leu*B6 *ara*-14 *pro*A2 *lac*Y1 *gal*K2 *xyl*-5 *mtl*-1 *rps*L20(Sm^R^) *gln*V44 *λ*^−^) and the cells were cultivated overnight at 37°C with shaking. Next day, overnight culture was used to inoculate fresh LB media to attain OD_600 nm_ = 0.25. DOXY (50 and 100 nM final concentrations), Na_2_S (10, 25 and 50 µM final concentrations) and Na_2_S/DOXY mixture (10/50, 25/50, 50/50 and 50/100 µM/nM final concentration ratios) were added to bacterial cultures that were then grown at 37 °C with shaking. Bacterial growth was monitored through the OD_600 nm_ measurement every hour within six hour period.

## 5. Conclusions

We present evidence that sulfide and polysulfides interact with tetracyclines and produce/scavenge free radicals. Some of the radical producing/scavenging properties display the bell-shaped behaviour that is dependent on time and/or concentration of the mixture components. Since H_2_S, and probably polysulfides, are endogenously produced in all organs, it may be suggested that some of the biological effects of tetracyclines are due to their interaction with H_2_S and/or polysulfides. Our results indicate that further studies of the biological effects of the H_2_S/polysulfide combination with tetracyclines may help to understand their possible mutual role in a “free radical signaling” and the combination may be useful in pathological states in which radicals play a negative role.

## Figures and Tables

**Table 1:** Hyperfine coupling constants of the BMPO spin adducts elucidated from the simulations of experimental spectra measured in the buffer solutions. ^•^BMPO-OOH and ^•^BMPO-OH were simulated based on two conformers.

BMPO-Adduct	*a*_N_, mT	*a*_H_^β^, mT	*a*_H_^γ^, mT
^•^BMPO-OH(1)	1.424 ± 0.008	1.27 ± 0.02	0.068 ± 0.005
^•^BMPO-OH(2)	1.41 ± 0.01	1.51 ± 0.01	0.06 ± 0.01
^•^BMPO-OOH(1)	1.33 ± 0.01	1.18 ± 0.01	–
^•^BMPO-OOH(2)	1.34 ± 0.01	0.97 ± 0.01	–

## Supplementary Materials

The following are available online. Figure S1: Time-dependent reduction of the ^•^cPTIO radical by Na_2_S_3_. Figure S2: EPR spectra of the ^•^BMPO adducts for Na_2_S_2_, Na_2_S and DOXY and their mutual combinations. Figure S3: Time-dependent reduction of the ^•^cPTIO radical by the studied compounds.
