# Supplementary material for: Sulfide (Na2S) and Polysulfide (Na2S2) Interacting with Doxycycline Produce/Scavenge Superoxide and Hydroxyl Radicals and Induce/Inhibit DNA Cleavage
Source: Molecules. 2019 Mar 22;24(6):1148. doi: 10.3390/molecules24061148 (PMC6470963; doi:10.3390/molecules24061148)
Supplement: Supplementary file 1 [file molecules-24-01148-s001.pdf]

Supplementary Materials

Sulfide (Na_2S) and polysulfide (Na_2S_2) interacting with doxycycline produce/scavenge superoxide and hydroxyl radicals and induce/inhibit DNA cleavage

Anton Misak[1], Lucia Kurakova[2], Eduard Goffa[3], Vlasta Brezova[4], Marian Grman[1], Elena Ondriasova[2], Miroslav Chovanec[3] and Karol Ondrias[1,]

[1] Institute of Clinical and Translational Research, Biomedical Research Center, University Science Park for Biomedicine, Slovak Academy of Sciences, 845 05 Bratislava, Slovak Republic
[2] Department of Pharmacology and Toxicology, Faculty of Pharmacy, Comenius University, 832 32 Bratislava, Slovak Republic
[3] Cancer Research Institute, Biomedical Research Center , University Science Park for Biomedicine, Slovak Academy of Sciences, 845 05 Bratislava, Slovak Republic
[4] Faculty of Chemical and Food Technology, Slovak University of Technology, 812 37 Bratislava, Slovak Republic

Correspondence: karol.ondrias@savba.sk, Tel.:+421-908577943

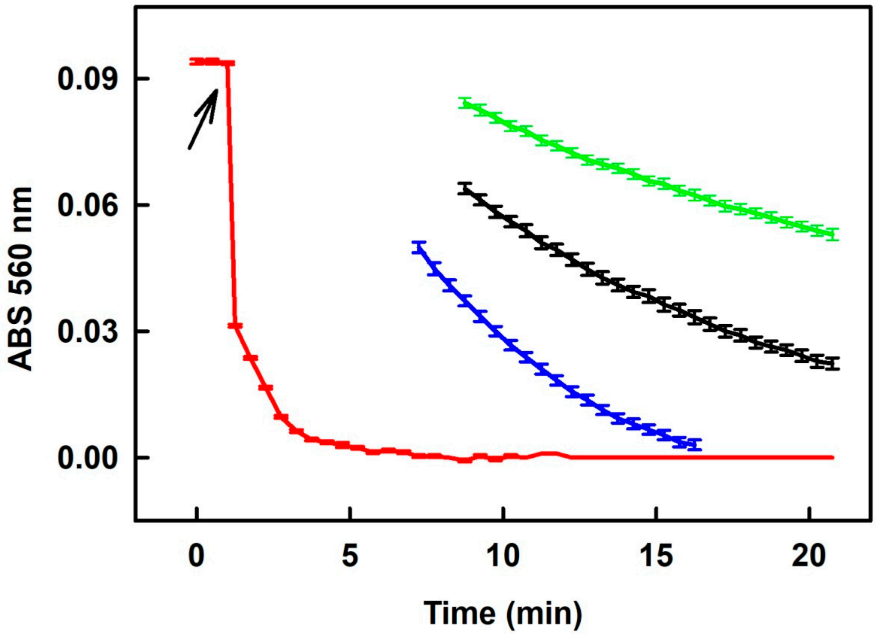

Figure S1. Time-dependent reduction of the •cPTIO radical by Na_2S_3. Reduction of the •cPTIO radical was detected as the decrease of ABS at 560 nm minus ABS at 730 nm (ABS 560 nm). Arrow indicates addition of 100 μM •cPTIO to 100 μM Na_2S_3 incubated 15 s (red), 20 min (blue), 40 min (black) and 70 min (green) in the buffer consisting of 100 mM sodium phosphate and 100 μM DTPA pH 7.4, at 37°C. Means ± SE; n = 3. Due to difficulties of background subtraction, the first (7-10 min) data for 20, 40 and 70 min incubation are not available.

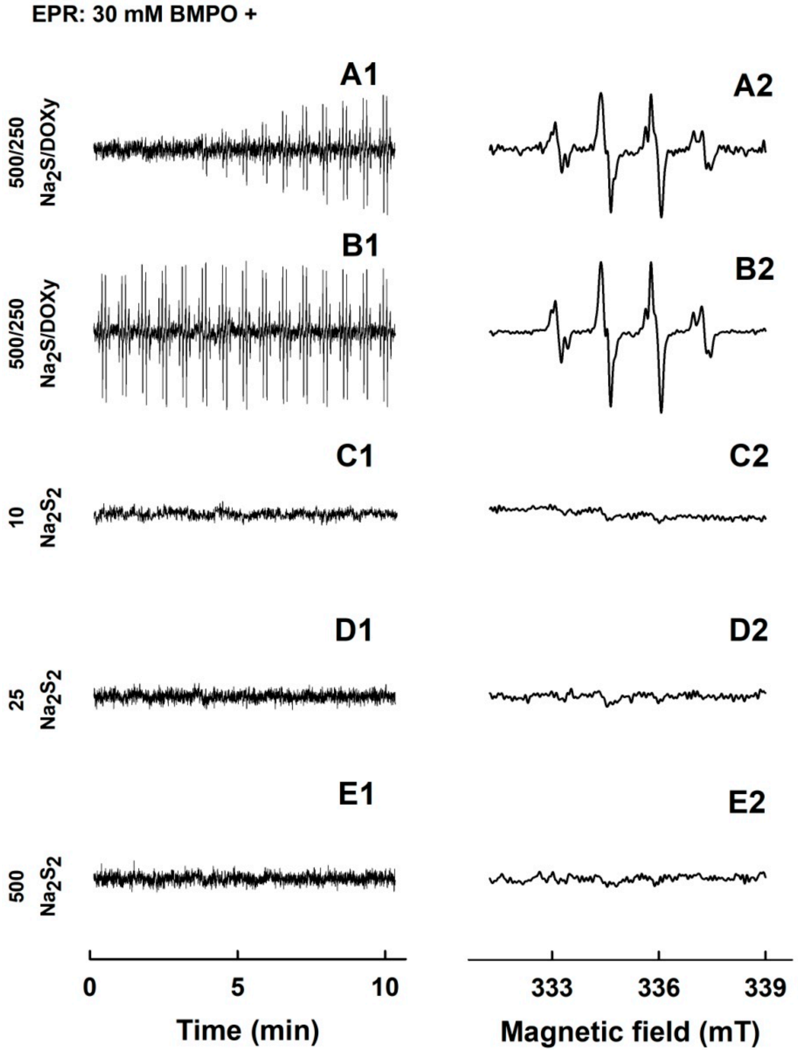

Figure S2. EPR spectra of the •BMPO adducts for Na_2S_2, Na_2S and DOXY and their mutual combinations. The sets of individual EPR spectra of the •BMPO adducts were monitored in 15 sequential scans, each 42 s (**A1**-**E1**), starting 110 ± 15 s after sample preparation. Fifteen EPR spectra were accumulated (**A2**-**E2**). The samples containing 30 mM •BMPO with 500/250 μM/μM Na_2S/DOXY monitored in 15 sequential scans (**A1**) and the continuation of the recording further 15 sequential scans (**B1**) over the next 10 minutes. •BMPO (30 mM) in the presence of 10 (**C1** and **C2**), 25 (**D1** and **D2**) and 500 μM Na_2S_2 (**E1** and **E2**). The intensities of the time-dependent EPR spectra (**A1**-**E1**) and detailed spectra (**A2**-**E2**) are comparable as they were measured under identical EPR settings (except for the spectra **B2**, which was multiply by 0.5). EPR spectra of the •BMPO spin-adducts were measured on a Bruker EMX spectrometer, X-band ~9.4 GHz, 335.15 mT central field, 8 mT scan range, 20 mW microwave power, 0.1 mT modulation amplitude, 42 s sweep time, 20.48 ms time constant, and 20.48 ms conversion time at 37°C.

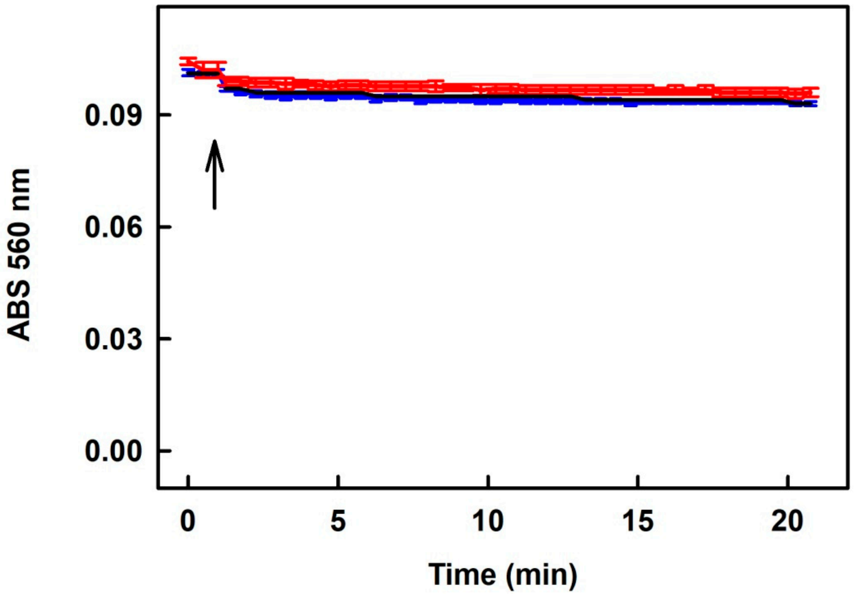

Figure S3. Time-dependent reduction of the •cPTIO radical by the studied compounds. Reduction of the •cPTIO radical was detected as decrease of ABS at 560 nm minus ABS at 730 nm (ABS 560 nm). Buffer: 100 mM sodium phosphate, 100 µM DTPA, pH 7.4, at 37°C. Arrow indicates addition of fusaric acid (400 µM, red) or norfloxacin (400 µM, blue) and control 5% DMSO (black) to 100/400 µM/µM •cPTIO/Na_2S. Means ± SE, n = 3.
